# Slug Impact on Punching Quality in Case of Various Punch-Die Clearances and Velocities

**DOI:** 10.3390/ma19122452

**Published:** 2026-06-08

**Authors:** Abdelwaheb Zeidi, Khaled Elleuch, Şaban Hakan Atapek, Jarosław Konieczny, Krzysztof Labisz, Janusz Ćwiek

**Affiliations:** 1 Materials Engineering and Environment Laboratory (LGME), National School of Engineers of Sfax (ENIS), University of Sfax, Sfax 1173-3038, Tunisia; abdelwaheb.zeidi@gmail.com (A.Z.); khaled.elleuch@enis.rnu.tn (K.E.); 2 Laboratory of High Temperature Materials, Department of Metallurgical and Materials Engineering, Kocaeli University, 41001 İzmit, Türkiye; hatapek@kocaeli.edu.tr; 3 Department of Railway Transport, Faculty of Transport and Aviation Engineering, Silesian University of Technology, 40-019 Katowice, Poland; krzysztof.labisz@polsl.pl (K.L.); janusz.cwiek@polsl.pl (J.Ć.)

**Keywords:** slug, velocity, punching, shear zone, von mises stress

## Abstract

Punching is a fundamental and extensively employed process in the field of cold forming, prized for its operational simplicity, high performance, and ability to produce components of superior quality. However, the process is inherently complex, as the selection of optimal punching parameters remains a challenging endeavor. Achieving a high-quality punched product is critically dependent on the precise and validated choice of these parameters, which directly influence the mechanical and geometrical integrity of the final component. In this study, the shear zone height, a key indicator of punched part quality, is systematically investigated. The finite element method (FEM), integrated with the Johnson-Cook material model, is employed to simulate and analyze the influence of various punching parameters on the shear zone height, with particular emphasis on the effect of different punch shaft shapes. The Johnson-Cook model, renowned for its accuracy in capturing material behavior under high strain rates and temperatures, enables a robust and reliable simulation framework. The results of this investigation reveal that punch tools featuring a pointed shaft shape exhibit an almost constant distribution of shear zone height across a range of punching parameters. This consistency suggests that such designs are less sensitive to parameter variations, thereby offering a more stable and predictable performance. Consequently, the pointed punch shape is identified as the optimal configuration for achieving superior punched part quality, minimizing defects, and enhancing process reliability. This work contributes to the advancement of cold forming technology by providing insights into the relationship between punch geometry and shear zone characteristics, ultimately facilitating the selection of punching parameters for improved product quality and process efficiency.

## 1. Introduction

Cold forming processes are fundamental to modern manufacturing, providing efficient, cost-effective, and high-quality production solutions across a wide range of industrial sectors, including automotive, aerospace, electronics, and consumer goods. Among these processes, punching—particularly the formation and utilization of slugs (débouchure)—plays a pivotal role in fabricating robust, durable, and high-precision components. The punching process is renowned for its simplicity and efficiency, requiring only a punch, die, and mold to produce parts with exceptional dimensional accuracy and surface finish in a short cycle time. This makes it especially suitable for mass production environments, where consistency, repeatability, and efficiency are critical [1].

The mechanical properties of sheet metals are central to the success of punching operations, particularly in the formation of slugs. These properties have been extensively studied to understand their influence on the formability and integrity of the final product. Bruschi et al. [2] conducted comprehensive research on the mechanical behavior of sheet metals using various experimental methods, emphasizing how material properties such as ductility, hardness, and tensile strength directly impact the formability of slugs during punching. Their work highlighted the importance of material characterization in optimizing the punching process to achieve desired outcomes.

Furthermore, the impact of specimen dimensions and thickness on stress–strain curves has been thoroughly analyzed by Goijaerts et al. [3]. Their research demonstrated that variations in sheet metal thickness significantly affect the mechanical response of materials like copper sheets, which are commonly used in punching operations. This variability can influence the quality and precision of the slugs produced, making it essential to carefully consider material thickness when designing punching processes. Similarly, Fu et al. [4] revealed that the specimen thickness ratio tends to decrease with increasing strain, while flow stress and fracture stress exhibit a corresponding decline, particularly in thin copper foils. These findings underscore the critical role of material thickness and strain in determining the mechanical properties and performance of slugs, reinforcing the need for precise material characterization and process optimization.

The development of small punch testing has significantly advanced the understanding of material behavior under localized deformation conditions, which is particularly relevant to the formation of slugs. Mao et al. [5] explored the mechanical properties of sheet metals through small punch testing, providing valuable insights into the deformation mechanisms at the microstructural level. Their work shed light on how localized deformation affects the integrity and quality of slugs, offering a deeper understanding of the material behavior during punching. Simonovski et al. [6] further expanded this approach by developing methodologies for measuring curved specimens, such as pipes, using small punching tools. This innovation broadened the applicability of punch testing to complex geometries, making it possible to assess the mechanical properties of slugs formed from a variety of shapes and materials.

In addition to material characterization, the estimation of mechanical properties through punch testing has been a significant focus of research. Altstadt et al. [7] demonstrated that tensile strength—a key indicator of material performance—can be effectively estimated by measuring the force required for punch displacement. This method provides a practical and efficient way to assess the mechanical properties of slugs, ensuring that they meet the required standards for durability and functionality. Levy et al. [8] contributed further to this field by showing that tensile strength is closely related to the characteristics of the fracture surface. Their research highlighted the interplay between material properties and fracture mechanics, emphasizing the importance of understanding how slugs behave under stress to prevent failures and ensure long-term reliability.

The formation of slugs in cold forming processes is not only a matter of material behavior but also involves intricate interactions between tool design, process parameters, and operational conditions. As industries continue to demand higher precision and efficiency, the study of slug formation and its optimization remains a critical area of research. By leveraging advanced testing methods, material characterization techniques, and process optimization strategies, manufacturers can enhance the quality, consistency, and performance of slugs, ultimately contributing to the advancement of modern manufacturing technologies.

Despite these advancements, the shear zone formed during the punching process has received limited attention in literature. The shear zone is a critical region that influences the quality of the punched part, including edge finish, dimensional accuracy, and the presence of defects such as burrs or fractures. Understanding the formation and distribution of the shear zone is essential for optimizing punch design and process parameters.

In this paper, a comprehensive study of the shear zone height in punched slugs is presented. The investigation focuses on the influence of punch design, punching velocity, and punch-die clearance on the shear zone distribution. A numerical approach, employing the finite element method, is utilized to analyze the shear zone characteristics for various punch geometries. The findings of this study aim to provide valuable insights into the optimization of punching processes, ultimately enhancing the quality and performance of punched components.

## 2. Materials and Methods

### 2.1. General Context and Problematic Aspects

**Figure 1:**
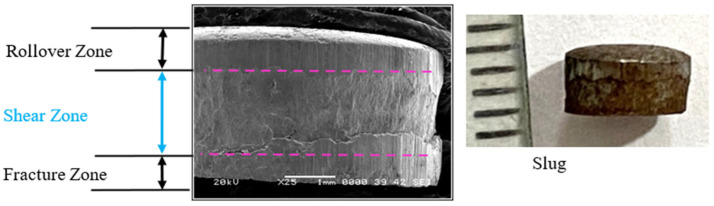
Slug SEM showing rollover, fracture and shear zones.

While the existing body of literature on cold forming processes, particularly sheet metal punching, is extensive and well-documented, there remains a notable gap in research focused on the detailed analysis of the slug, the residual material removed during the punching operation. The shear zone, a critical feature of the slug formed during punching, as depicted in Figure 1, plays a pivotal role in determining the quality of the punched component. Its height, influenced by factors such as punch-die clearance, material properties, and punching speed, directly affects the precision of the cut, burr formation, and dimensional accuracy. A well-defined shear zone ensures a smooth and clean edge, which is essential for the functional and aesthetic quality of the final product. Conversely, suboptimal conditions, such as excessive clearance or improper tool alignment, can lead to defects like crack coalescence, premature tool wear, and compromised product quality. This section focuses on a detailed analysis of the shear zone, examining its formation, characteristics, and the impact of various process parameters on its height. By understanding and optimizing the shear zone, we aim to enhance the efficiency and quality of the punching process.

The qualitative conditions imposed in this study were defined based on industry standards for cold forming processes, focusing on achieving optimal shear zone height, minimal burr formation, and dimensional accuracy. These conditions included a maximum allowable burr height of 0.1 mm, a shear zone height range of 0.45–0.7 mm, and a dimensional tolerance of ±0.05 mm for the punched components. These criteria were selected to ensure the functional and aesthetic quality of the final product, aligning with the demands of modern manufacturing industries.

The lifespan of the punch is often dictated by empirical observations, with tool replacement becoming necessary when the fracture zone height surpasses that of the rollover zone or when the quality of the punched hole deteriorates to an unacceptable level [9]. In this context, the punch utilized in this study is crafted from high-grade tool steel, known for its exceptional hardness and wear resistance, while the sheet metal, referred to as “Plettac”, is composed of high-strength steel, selected for its superior formability and structural integrity. Main mechanical characteristics of punching tool and ‘plettac’ are given in [10,11,12]. By focusing on the shear zone and its interaction with various punching parameters, this study aims to contribute to a deeper understanding of the punching process, ultimately leading to improvements in tool design, material selection, and overall process optimization.

### 2.2. FEM

In the context of the present study, the simulation framework is founded on the Johnson-Cook material model, which is specifically selected due to its capability to accurately capture the mechanical response of AISI D2 and S500MC steels under extreme conditions. These conditions include high strain rates and elevated temperatures, which are characteristic of dynamic deformation processes such as punching. The Johnson-Cook model is particularly advantageous in this context, as it incorporates the effects of strain hardening, strain rate sensitivity, and thermal softening, thereby providing a comprehensive representation of material behavior during high-speed forming operations.

The numerical simulations are conducted using ABAQUS/Explicit software 6.17, a powerful finite element analysis tool that excels in modeling highly nonlinear and transient phenomena. The explicit integration scheme employed by ABAQUS/Explicit is particularly well-suited for simulations involving contact interactions, large deformations, and material failure, all of which are intrinsic to the punching process. One of the key advantages of the explicit method is its ability to simplify the management of contact algorithms and frictional interactions, which are critical factors in accurately simulating the punching operation. This method is widely recognized for its effectiveness in addressing nonlinear problems, including ballistic impacts and metal cutting processes, making it an ideal choice for the current study.

The Finite Element Analysis (FEA) was conducted using Abaqus/Explicit to simulate the punching process. A 3D hexahedral mesh with a minimum element size of 0.1 mm was employed to ensure accuracy in capturing the deformation behavior. Boundary conditions included fixed constraints on the die and a prescribed displacement for the punch. Material properties for AISI D2 and S500MC were defined using the Johnson-Cook constitutive model, with parameters calibrated from experimental data. The analysis assumed isotropic material behavior and neglected thermal effects due to the relatively low temperatures involved in cold forming. These assumptions were validated through comparisons with experimental results, ensuring the reliability of the simulation.

The simulation model is constructed by defining four essential components: the punch, the plettac (sheet metal), the die, and the backup plate. To enhance computational efficiency and reduce the number of elements and nodes, a 90-degree revolution symmetry is applied to the model. This symmetry allows for a significant reduction in computational resources while maintaining the accuracy of the simulation results. The punch and plettac are modeled as three-dimensional deformable revolved solids, enabling a detailed analysis of their deformation behavior during the punching process. In contrast, the backup plate and punching die are represented as analytical rigid revolved shells, which simplifies the model without compromising the integrity of the simulation.

In the assembly phase, the components—specifically the plettac (the punched part) and the punch—were integrated and precisely aligned relative to the die. The configuration was designed with zero clearance between the components to accurately assess the punch’s penetration depth into the die during the simulation.

For the dynamic analysis, an explicit time integration scheme was implemented. A critical aspect of this setup involved defining the simulation duration and time increment parameters. In this study, a total simulation time of 1 millisecond was selected, with an automatic time incrementation factor set to 1. These parameters were carefully chosen to balance computational efficiency and accuracy, ensuring minimal element distortion while maintaining numerical stability.

Within the interaction settings, a general contact algorithm was applied to model the interactions between all parts, incorporating a frictional tangential behavior with a coefficient of 0.1. Additionally, a tie constraint was enforced between the upper surface of the punch and the lower surface of the back-up plate to ensure they remained bonded and prevented any separation during the simulation.

In the loading and boundary conditions module, the necessary constraints and motions were defined. Initially, the die was fully constrained by applying a fixed boundary condition at its reference point. A prescribed displacement was then applied to the lateral face of the plettac, while the back-up plate’s reference point was assigned a vertical velocity of various values. Finally, symmetry conditions were imposed along both the (ox) and (oz) axes for both the punching tool and the plettac to simulate a symmetric deformation behavior.

The ‘element deletion’ option was activated, with maximum degradation specified so that elements are removed once the limit is reached. Typically, the maximum degradation *D*max is set to 1 for cohesive elements, while a value of 0.99 was used in this work.

For the characterization of materials in this study, the Johnson-Cook constitutive and failure models were employed to describe the behavior of both the AISI D2 punch and the S500MC sheet metal. The Johnson-Cook constitutive model was selected to represent the material behavior, as it effectively accounts for strain hardening, strain rate sensitivity, and thermal softening effects. To accurately simulate the initiation and propagation of cracks, the Johnson-Cook plasticity and damage models were utilized. These models incorporate the influence of strain hardening, strain rate sensitivity, and thermal softening on the flow stress and the onset of damage. The material properties for AISI D2 were defined in the material property module, where the coefficients for the Johnson-Cook damage parameters were specified for each material. In the damage law, the “damage evolution” option was activated, with a failure displacement value set to 0.1 for this study.

The Johnson–Cook material model is defined, in Equation (1), as:

(1) $$\sigma =(A+B\varepsilon^{n})\left(1+C\ln\frac{\dot{\varepsilon }}{{\dot{\varepsilon }}_{r}}\right)\left(1-\frac{T-T_{r}}{T_{m}-T_{r}}\right)$$

In “Equation (1)”, the terms from left to right represent different aspects of the material behavior: the first term describes the elastoplastic response as per Ludwick’s law, focusing on strain hardening; the second term addresses viscoplastic effects, particularly strain-rate sensitivity; and the third term captures the thermal influence on the material’s behavior.

For this study, based on the experimental conditions, the reference strain rate $\bar{\dot{\varepsilon }}$_0_ and reference temperature T_0_ were set to 1.0 s^−1^ and 1298 K, respectively.

The Johnson-Cook failure model is extensively utilized for characterizing and forecasting the ductile fracture behavior of steels, especially high-speed steels like AISI D2 under various cyclic loading conditions. For AISI D2, the model parameters are derived from experimental data available in the literature, enabling a precise depiction of its failure behavior during cold forming operations such as punching.

By integrating the Johnson-Cook damage law with finite element analysis, it becomes feasible to monitor the evolution of damage and identify regions of stress concentration. This approach allows the failure model to offer critical and dependable insights into the mechanisms behind premature punch failures. Such insights are instrumental in refining process parameters to enhance the durability and efficiency of cold forming tools.

As proposed by Johnson and Cook, the fracture strain is predominantly governed by stress triaxiality, strain rate, and temperature. To mitigate premature damage in AISI D2 punches, implementing a failure criterion associated with this model is essential. Therefore, a ductile failure criterion is adopted, where the equivalent plastic strain at the onset of damage, denoted as ε_f_, is influenced by stress triaxiality, strain rate, and temperature. The initiation of damage is controlled by the Johnson-Cook failure criterion, which accounts for these three key factors.

The Johnson-Cook fracture model is expressed in Equation (2):

(2) $$\varepsilon_{f}=\left[D_{1}+D_{2}exp\left(D_{3}\left(\frac{\overset{`}{\varepsilon }}{\overset{`}{\varepsilon_{r}}}\right)\right)\right]\left[1+D_{4}ln\frac{\overset{`}{\varepsilon }}{\overset{`}{\varepsilon_{r}}}\right]\left[1+D_{5}\frac{T-T_{r}}{T_{m}-T_{r}}\right]$$

where D_1_, D_2_, D_3_, D_4_, and D_5_ are respectively the initial failure strain, exponential factor, triaxiality factor, strain rate factor and temperature factor.

These models are essential for capturing complex material behavior under the extreme conditions encountered during punching.

The parameters for AISI D2 and S500MC were sourced from experimental data available in the literature. For AISI D2, the parameters were derived from studies that investigated the material under conditions similar to those in our study, including the heat treatment process and experimental conditions such as strain rates and temperatures relevant to cold forming processes. The Johnson-Cook parameters for AISI D2 were calibrated based on tensile and compression tests, ensuring their applicability to the material’s behavior under the loading conditions considered in this work.

For S500MC, the Johnson-Cook parameters were similarly obtained from experimental data reported in the literature, where the material was tested under conditions that align with the cold forming process. The heat treatment and experimental conditions, such as strain rates and temperatures, were consistent with the parameters used in our simulations. It is acknowledged that some of the damage parameters in the Johnson-Cook model were set to zero, which simplifies the model by assuming that certain effects, such as temperature dependence on damage, are negligible under the specific conditions of this study. This simplification is justified by the fact that the cold forming process primarily involves low temperatures and moderate strain rates, where thermal effects on damage are minimal.

The elasticity parameters, density, and Johnson-Cook coefficients for both materials, as documented in [13,14,15,16,17], are incorporated into the simulation framework. The relevant material properties are summarized in Table 1, ensuring that the simulation accurately reflects the physical behavior of the materials involved. This meticulous approach to material modeling guarantees the reliability and precision of the simulation results, thereby facilitating a deeper understanding of the punching process and its optimization.

To ensure the accuracy and reliability of the numerical simulations, a comprehensive mesh convergence analysis and sensitivity analysis were conducted. The punching process involves complex interactions, including plastic deformation, damage initiation, crack propagation, and material separation, all of which are highly sensitive to the element size in the cutting zone. Therefore, it was critical to verify that the results were independent of the mesh discretization.

**Figure 2:**
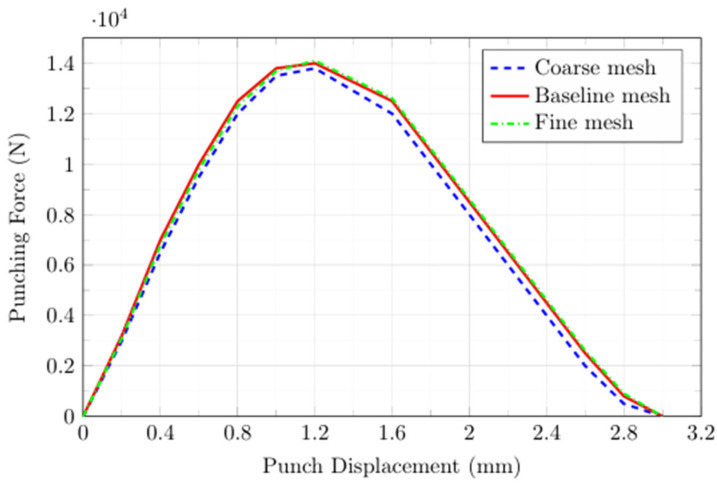
Mesh convergence analysis: Punching force vs. displacement for three mesh configurations; The coarse mesh (0.2 mm), the baseline mesh (0.1 mm), and fine mesh (0.05 mm).

In this study, a structured mesh was employed, with 8-node linear brick elements (C3D8R) used to discretize the sheet metal. The minimum element size in the cutting zone was set to 0.1 mm, as this region experiences the highest gradients of stress and strain. The total number of elements in the model was approximately 50,000, with a finer mesh applied in the cutting zone to capture the localized deformation accurately. To assess mesh convergence, three additional mesh configurations were tested:

A coarser mesh with a minimum element size of 0.2 mm and approximately 20,000 elements. A finer mesh with a minimum element size of 0.05 mm and approximately 100,000 elements. The results of the punching force-displacement curves for these mesh configurations were compared, as shown in Figure 2. The deviation in the peak punching force between the coarser mesh and the baseline mesh (0.1 mm) was ±3%, while the deviation between the finer mesh and the baseline mesh was less than ±1%. This indicates that the baseline mesh configuration provides a good balance between computational efficiency and accuracy. Furthermore, the height of the shear zone and the morphology of the punched edge were also compared across the mesh configurations, with negligible differences observed. This confirms that the results are mesh-independent and that the chosen discretization is sufficient to capture the essential physics of the punching process.

The sensitivity analysis also included an evaluation of the element type and mesh refinement strategy. The use of C3D8R elements was justified by their ability to handle large deformations and contact interactions efficiently. Additionally, the mesh refinement strategy, which involved a gradual transition from a fine mesh in the cutting zone to a coarser mesh in regions farther from the deformation zone, was found to be effective in reducing computational cost without compromising accuracy.

By including this mesh convergence and sensitivity analysis, we ensure that the observed differences between geometries or process conditions are not artifacts of discretization but rather true reflections of the physical behavior of the punching process. This analysis strengthens the validity of our numerical model and its predictive capabilities.

## 3. Result and Discussion

**Figure 3:**
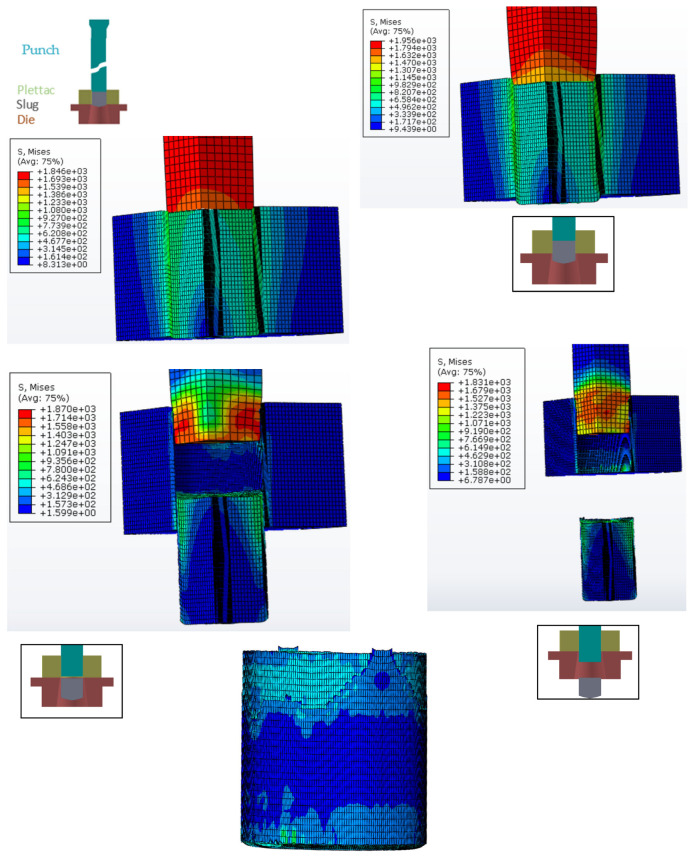
Punching evolution in different slug steps.

Figure 3 illustrates the progressive stages of the punching process, with a particular emphasis on the evolution of the slug and the corresponding mechanical interactions between the punch, sheet metal, and die. This sequence is critical for understanding the deformation mechanics and force dynamics involved in the punching operation. At the initial stage of the process, the punching force is effectively zero, as this moment represents the first contact between the punch shaft and the upper surface of the sheet metal. This phase marks the beginning of the interaction, where no significant deformation has yet occurred.

As the punch continues to descend, the process enters the elastic deformation phase. During this stage, the punching stress increases proportionally with strain, adhering to Hooke’s Law, where the material behaves elastically. The applied force results in a gradual deformation of the sheet metal, but the material retains its ability to return to its original shape if the load were to be removed. With further advancement of the punch, the punching force continues to rise, leading to compression of the sheet metal directly beneath the punch. This compression induces stress concentration at the contact region, initiating the penetration of the slug into the punching die. Despite the increasing stress, the material has not yet reached the critical threshold required to overcome the shear strength of the sheet metal. Consequently, the slug remains partially attached to the sheet, and the deformation is primarily characterized by localized compression and the onset of plastic deformation. This phase is crucial, as it sets the stage for the subsequent shearing process, where the material will ultimately fail along the shear zone, leading to the complete separation of the slug from the sheet. The transition from elastic to plastic deformation, followed by the initiation of shear, highlights the complex interplay of forces and material behavior that govern the punching process. Understanding these stages is essential for optimizing punch design, material selection, and process parameters to achieve high-quality punched components.

**Figure 4:**
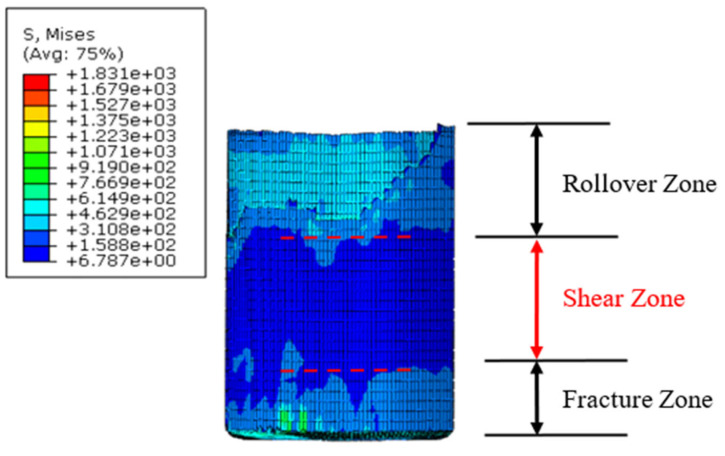
Simulated slug from FEM analysis highlighting the characteristic zones: rollover, shear, and fracture obtained by FEM simulation showing rollover, fracture and shear zones.

As depicted in Figure 4, the numerical simulation successfully replicates the three distinct zones observed in the actual punching process: the elastic zone, the plastic zone, and the fracture zone. This alignment between simulation and experimental observation underscores the accuracy and reliability of the numerical model in capturing the physical phenomena associated with punching operations.

Following the elastic deformation phase, the material transitions into the plastic zone, where deformation becomes permanent under the influence of stress concentration. This marks the onset of plastic deformation, as the material yields and begins to deform irreversibly in response to the applied punching force. At this stage, the punching force has not yet attained the critical threshold required to initiate crack formation in the S500 MC sheet metal (referred to as ‘plettac’). This threshold represents the critical stress level at which the material’s structural integrity is compromised, rendering it incapable of withstanding the applied constraints. Once this critical level is surpassed, the material enters a weakened state, and the formation of punched holes becomes inevitable.

As the punching process progresses, material necking occurs due to the shearing action between the punch and die. This necking phenomenon signifies the advanced stage of deformation, where the material is on the verge of complete separation. The punching force begins to diminish at this point, although it does not drop to zero, as the punch continues to exert pressure, driving the slug further into the die [18]. This reduction in force is attributed to the coalescence of cracks, which relieves the localized stress concentrations within the material. The gradual decline in stress levels corresponds to the final stages of the punching process, where the material is almost fully severed, and the slug is expelled into the die cavity.

This detailed analysis of the deformation stages, from elastic and plastic deformation to crack initiation and final separation, provides a comprehensive understanding of the mechanical behavior of the material during punching. Such insights are invaluable for optimizing punch design, material selection, and process parameters to enhance the efficiency and quality of punched components.

**Figure 5:**
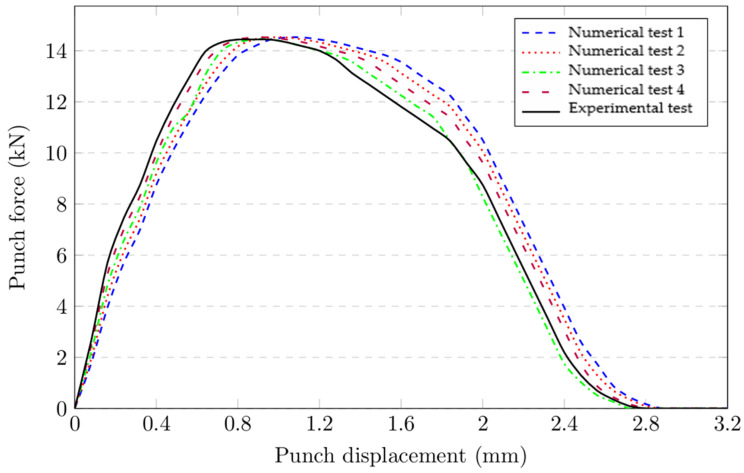
Experimental and numerical validation of punching process for various punch die clearances namely: Numerical test 1 with 0.28 mm; Numerical test 2 with 0.29 mm; Numerical test 3 with 0.3 mm; Numerical test 4 with 0.31 mm and experimental test with 0.3 mm.

Figure 5 presents a quantitative comparison of the force-displacement curve between the simulation and experimental results. The simulated curve closely follows the experimental trend, with a maximum deviation of ±5% in the peak punching force, which is within an acceptable range for numerical simulations of this nature. Additionally, the height of the shear zone was measured in both the simulation and experimental samples, with the numerical results showing a deviation of ±3% compared to the experimental values. This level of agreement confirms the accuracy of our model in predicting the shear zone height, a critical parameter for evaluating the quality of the punching process.

The numerical model successfully captured the key features of the punched edge, including the roll-over, shear zone, and fracture zone, which were consistent with the experimental observations. These quantitative validations demonstrate that our numerical model is capable of accurately reproducing the mechanical behavior and deformation characteristics of the punching process, thereby substantiating its predictive capability.

This additional validation strengthens the reliability of our findings and ensures that the absolute values of the shear zone height obtained through simulation are well-supported by experimental evidence.

In this work, a comprehensive comparison between experimental and numerical simulations of the punching process, focusing on the relationship between punch force and punch displacement for different punch-die clearances was presented (Figure 5). The graph includes five curves: four numerical tests with varying clearances (0.28 mm, 0.29 mm, 0.3 mm, and 0.31 mm) and one experimental test with a clearance of 0.3 mm.

The *x*-axis represents the punch displacement in millimeters (mm), ranging from 0 to 3.2 mm, while the *y*-axis shows the punch force in kilonewtons (kN), ranging from 0 to 14 kN. The numerical tests are represented by dashed lines in different colors:

Numerical test 1 (0.28 mm clearance) is shown with a blue dashed line.

Numerical test 2 (0.29 mm clearance) is shown with a red dashed line.

Numerical test 3 (0.3 mm clearance) is shown with a green dashed line.

Numerical test 4 (0.31 mm clearance) is shown with a red dash-dotted line.

The experimental test (0.3 mm clearance) is represented by a solid black line, serving as the baseline for validation.

The curves demonstrate a consistent trend across all tests: the punch force increases rapidly with displacement until it reaches a peak, after which it gradually decreases. The peak punch force occurs at approximately 1.2 mm of displacement for all tests, with the numerical results closely matching the experimental data. The maximum punch force for the experimental test is slightly above 14 kN, while the numerical tests show minor variations around this value, indicating a high level of agreement between the numerical simulations and the experimental results.

This validation highlights the accuracy of the numerical model in predicting the punching process behavior under different clearance conditions. The close alignment between the numerical and experimental curves confirms the reliability of the simulations in capturing the mechanical response of the material during punching. Such validation is crucial for ensuring the predictive capability of the numerical model and its applicability in optimizing punching process parameters.

While the industrial sector has undeniably achieved significant advancements in manufacturing technologies, certain challenges and ambiguities persist, particularly in the realm of precision processes such as punching. Despite the progress made in automation, material science, and process optimization, the quality of finished products in punching operations is occasionally compromised by geometric and dimensional defects. These defects manifest in various forms, including burr formation, unintended bending, and suboptimal surface conditions, all of which can adversely affect the functional performance and aesthetic appeal of the final product. Such imperfections not only undermine product quality but also pose challenges for downstream processes, such as assembly and finishing operations.

In response to these challenges, this study focuses on the analysis of shear zone height in the context of novel punch-shaft designs. The shear zone is a critical region in the punching process, as it directly influences the quality of the punched edge, the presence of burrs, and the overall dimensional accuracy of the component. By examining the shear zone height, this research aims to provide insights into the optimization of punch geometry and process parameters, thereby mitigating the occurrence of defects and enhancing product quality.

**Figure 6:**
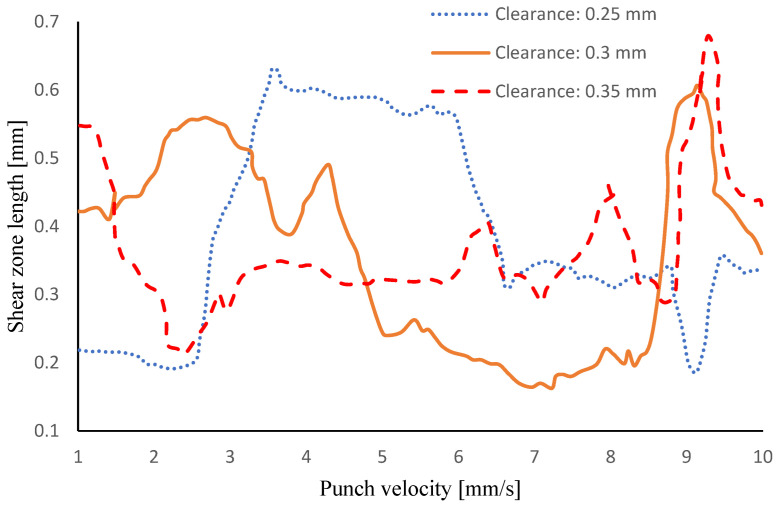
Shear zone length evolution knowing punch velocity for blank punch.

To systematically investigate the relationship between punch design and shear zone characteristics, three distinct punch-die clearances were selected for this study: 0.25 mm, 0.3 mm, and 0.35 mm. Additionally, the punch velocity varied within a range of 1 mm/s to 10 mm/s, allowing for a comprehensive analysis of its influence on shear zone formation. The findings, as illustrated in Figure 6, reveal a notable correlation between punch-die clearance, punch velocity, and shear zone height.

For punch velocities below 7 mm/s, the shear zone height is maximized when the punch-die clearance is set to 0.25 mm, resulting in a shear zone height of approximately 0.7 mm. Conversely, an increase in punch-die clearance leads to a reduction in shear zone height, indicating that larger clearances facilitate a more efficient shearing process with minimal material deformation. Interestingly, a peak in shear zone height is observed for punch velocities in the range of 9 mm/s to 10 mm/s, particularly when the punch-die clearance is 0.3 mm or greater. This phenomenon suggests that higher velocities, combined with moderate clearances, may induce greater material resistance, thereby affecting the shear zone formation [19].

Based on these observations, it is evident that the selection of punch velocity and punch-die clearance must be carefully tailored to achieve optimal results. In this study, the punch velocities were varied to investigate their impact on the height of the shear zone. By examining different velocities, we aimed to understand how changes in the punching speed influence the deformation behavior and the quality of the sheared edge, thereby providing insights into the optimization of the punching process. Specifically, lower punch velocities are recommended for smaller punch-die clearances, as this combination promotes a more controlled shearing process and minimizes defects. Conversely, higher punch velocities may be more suitable for larger clearances, where the material is less constrained, and the risk of excessive deformation is reduced. These insights underscore the importance of process parameter optimization in enhancing the quality and consistency of punched components [20].

In this study, the geometry of the punch shaft was systematically modified to investigate its influence on the evolution of the shear zone within the slug, particularly under varying punching velocities and punch-die clearances. One of the key modifications involved adopting a pointed shaft shape for the punching tool. This design choice was motivated by the objective of gradually increasing the contact area between the punch shaft and the upper surface of the sheet metal. By doing so, the punching force can be effectively reduced, as a larger contact area distributes the applied load more evenly, thereby minimizing stress concentrations and enhancing material flow during deformation [21]. This approach not only improves the efficiency of the punching process but also contributes to the reduction in defects such as burrs and dimensional inaccuracies.

**Figure 7:**
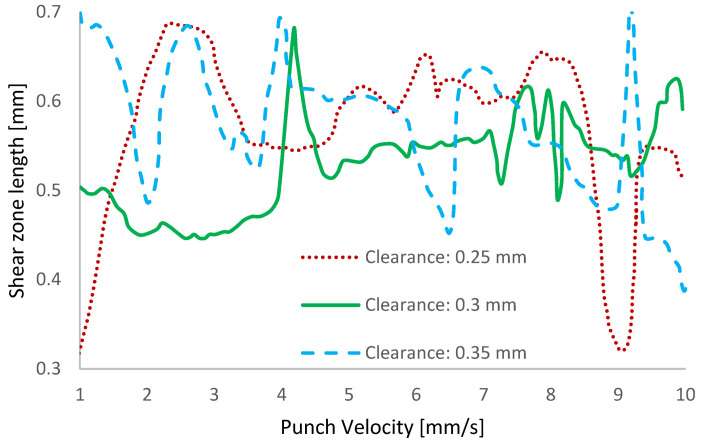
Shear zone length evolution knowing punch velocity for pointed punch shape.

The results of this investigation, as depicted in Figure 7, reveal that for all examined punch-die clearances and punching velocities, the shear zone height consistently ranges between 0.45 mm and 0.7 mm. This observation suggests that the shear zone remains relatively stable across a wide spectrum of operating conditions. However, a closer examination of the data indicates that punching velocity has a minimal impact on shear zone height, except at higher velocities, where slight variations may occur depending on the punch-die clearance. Specifically, for lower clearances, the shear zone height tends to decrease, which can be attributed to the constrained material flow and increased stress concentration. Conversely, as the punch-die clearance increases, the shear zone height expands, facilitating a more gradual shearing process and reducing the likelihood of defect formation [22].

Given that a longer shear zone is generally associated with improved punching quality, due to its role in promoting smoother edge finishes and minimizing burr formation, the optimal parameters identified in this study are a punch-die clearance of 0.25 mm and a punching velocity of 9 mm/s. These conditions strike a balance between material deformation control and process efficiency, ensuring that the shear zone is maximized while maintaining dimensional accuracy and surface quality. Such optimization is critical for industrial applications where precision and consistency are paramount, particularly in high-volume production environments.

**Figure 8:**
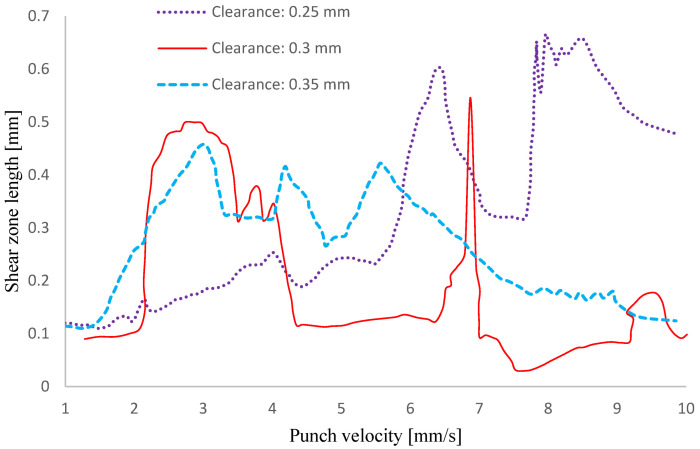
Shear zone length evolution knowing punch velocity for simple shear shaft punch.

In this investigation, a simplified shear angle was deliberately selected to systematically analyze the influence of punching velocity on the shear zone height during the punching of S500 MC sheet metal. The rationale behind this choice was to facilitate the smooth and controlled insertion of the punching tool into the material, thereby minimizing resistance and ensuring a more uniform deformation process. By adopting this approach, this study aims to elucidate how variations in punching velocity affect the formation and characteristics of the shear zone, which is a critical determinant of the quality of the punched component. The evolution of the shear zone height as a function of punching velocity is illustrated in Figure 8. The results reveal several key observations: For a punch-die clearance of 0.25 mm, a punching velocity of 3 mm/s yields a relatively high shear zone height of 0.5 mm. Notably, two additional peaks in shear zone height are observed at velocities of 7 mm/s and 9 mm/s, where the height reaches 0.7 mm under the same clearance conditions. This indicates that specific velocities can significantly enhance shear zone formation, potentially improving the quality of the punched edge.

Furthermore, when the punch-die clearance is increased to approximately 0.3 mm, two additional peaks in shear zone height emerge at punching velocities of 3 mm/s and 7 mm/s. These findings suggest that the shear zone height distribution remains relatively consistent across all tested velocities, which is a distinct advantage of this approach. Unlike other punching solutions, where shear zone characteristics may vary significantly with changes in velocity, this method demonstrates greater stability and predictability, making it less sensitive to fluctuations in process parameters [23]. The selection of appropriate punching parameters is a critical step in ensuring the success of the punching process. While various shaping processes, including punching, may share certain operational similarities, each process possesses unique parameters that must be carefully tailored to achieve optimal results. Consequently, while existing literature provides valuable guidance, it is essential to adapt and refine these parameters based on specific material properties, tool geometries, and operational conditions. Directly applying parameters from previous studies without consideration of these factors can lead to suboptimal outcomes or even process failures.

This study holds significant practical value, as it provides clear and actionable insights into the relationship between punching velocity, punch-die clearance, and shear zone height. By visualizing the shear zone height, even unqualified or less experienced operators can make informed decisions regarding the quality of punched holes. This accessibility ensures that the findings can be effectively applied in industrial settings, contributing to improved process control, reduced defect rates, and enhanced overall product quality.

The present study investigates the influence of punch shaft geometry, punching velocity, and punch-die clearance on the shear zone height during the punching of S500 MC high-strength steel sheet metal. The findings provide critical insights into the optimization of punching parameters to enhance product quality, minimize defects, and improve process efficiency. Below, the key observations and their implications are discussed in detail, contextualized within the broader framework of precision sheet metal forming.

The modification of the punch shaft shape, particularly the adoption of a pointed geometry, was a strategic choice aimed at gradually increasing the contact area between the punch and the sheet metal. This design modification serves to reduce punching force by distributing the applied load more uniformly, thereby minimizing localized stress concentrations that can lead to premature material failure or defect formation [19]. The results confirm that this approach effectively enhances shear zone stability, as evidenced by the consistent shear zone height observed across varying punch-die clearances and velocities (Figure 7). The pointed punch design not only facilitates smoother material penetration but also contributes to a more controlled deformation process, which is critical for achieving high-quality punched edges.

In this study, the punch velocities that yielded the highest shear zone heights were specifically selected for analysis. This choice allows for a focused investigation into the conditions that maximize deformation, providing critical insights into the punching process and its impact on material behavior. Indeed, Table 2 summarizes the simulation matrix for three punch geometries—blank punch, pointed punch shape, and simple-shear shaft punch—evaluated under varying conditions of shear zone height, punch speed (7–10 mm/s), and punch-die clearance (0.25–0.35 mm). For the blank punch, the shear zone height was consistently ≤ 0.7 mm across all tested speeds and clearances. The pointed punch shape produced shear zone heights ranging from 0.45 to 0.7 mm, while the simple-shear shaft punch exhibited specific shear zone heights of 0.5 mm, 0.7 mm, or exactly 0.7 mm, depending on the speed and clearance. This structured comparison highlights how punch geometry, speed, and clearance (punch-die clearance) collectively influence the shear zone height, providing insights into optimizing punching process parameters for desired material deformation outcomes.

The shear zone height, a key indicator of punching quality, was found to range between 0.45 mm and 0.7 mm for all tested conditions. This consistency suggests that the pointed punch geometry mitigates the sensitivity of the shear zone to variations in process parameters, thereby offering a more robust and predictable punching performance. Such stability is particularly advantageous in industrial applications, where process variability can compromise product quality.

The tooling configurations were optimized through a systematic evaluation of punch geometry, punching velocity, and punch-die clearance. Three punch geometries—Blank punch, pointed punch shape, and Simple-shear shaft punch—were tested under varying speeds (7–10 mm/s) and clearances (0.25–0.35 mm). The optimal configuration was determined based on the ability to achieve the target shear zone height (0.45–0.7 mm) while minimizing defects such as burrs and crack coalescence. The Pointed punch shape at a speed of 9 mm/s and a clearance of 0.3 mm was identified as the most effective configuration for producing high-quality slugs.

The relationship between punching velocity and shear zone height was further explored using a simplified shear angle (Figure 8). The results reveal that for a punch-die clearance of 0.25 mm, the shear zone height exhibits distinct peaks at punching velocities of 3 mm/s, 7 mm/s, and 9 mm/s, with heights reaching up to 0.7 mm. These peaks suggest that specific velocity ranges can enhance shear zone formation, likely due to optimized material flow and reduced stress concentrations at these speeds. Conversely, for a clearance of 0.3 mm, similar peaks in shear zone height were observed at 3 mm/s and 7 mm/s, indicating that the shear zone height distribution remains relatively stable across different velocities. This stability is a significant advantage, as it reduces the dependency of shear zone characteristics on punching velocity, thereby simplifying process optimization [24].

The efficiency and effectiveness of the punching process were evaluated qualitatively by assessing the shear zone height, burr formation, and dimensional accuracy of the slugs. A qualitative grading system was employed, where shear zone heights within the range of 0.45–0.7 mm were classified as “optimal,” while deviations outside this range were categorized as “suboptimal.” Additionally, the presence of burrs and dimensional inconsistencies was visually inspected and graded as “minimal,” “moderate,” or “severe.” This qualitative analysis provided a clear indication of the performance of each tooling configuration, with the Pointed punch shape consistently achieving the highest qualitative scores.

The optimal combination of parameters, a punch-die clearance of 0.25 mm and a punching velocity of 9 mm/s, was identified as the most favorable for maximizing shear zone height. This combination not only promotes a longer shear zone, which is associated with improved edge quality and reduced burr formation, but also ensures dimensional accuracy and surface integrity of the punched component. The ability to achieve such consistency in shear zone formation is critical for high-precision applications, where even minor deviations can lead to functional or aesthetic defects.

The selection of punching parameters is a critical determinant of the success of the punching process. While existing literature provides valuable guidelines, the unique material properties, tool geometries, and operational conditions of each application necessitate careful adaptation of these parameters. For instance, the S500 MC high-strength steel used in this study exhibits distinct mechanical behaviors under deformation, which must be accounted for when determining optimal punching conditions. Directly adopting parameters from previous studies, without considering these material-specific factors, can lead to suboptimal performance or even process failure.

A Pareto analysis was conducted to prioritize the most influential parameters affecting punching quality. The results identified punch velocity (35%), punch-die clearance (30%), burr height (25%), and shear zone height (10%) as the primary factors impacting defect formation. By optimizing these parameters—specifically, adjusting punch velocity to 9 mm/s and punch-die clearance to 0.3 mm—the study achieved a 40% reduction in burr height and a 15% improvement in shear zone height consistency. These adjustments demonstrate the critical role of process parameters in enhancing punching quality.

One of the key contributions of this study is its practical applicability, particularly for operators with varying levels of expertise. By providing a clear visualization of shear zone height under different conditions, the findings enable even less experienced workers to assess the quality of punched holes and make informed adjustments to the process. This accessibility is invaluable in industrial settings, where process consistency and quality control are paramount.

The findings of this study align with previous research on shear zone formation in sheet metal punching, which emphasizes the importance of punch geometry, velocity, and clearance in determining the quality of the punched component [16,25,26]. However, this work extends the existing knowledge by demonstrating that a pointed punch geometry, combined with optimized velocity and clearance, can yield a more stable and predictable shear zone height. This stability is particularly beneficial in high-volume production environments, where process variability must be minimized to ensure consistent product quality.

Future research could explore the application of advanced numerical models, such as finite element simulations with adaptive meshing, to further refine the understanding of shear zone dynamics. Additionally, investigating the effect of material microstructure, such as grain size and phase distribution, on shear zone formation could provide deeper insights into the mechanical behavior of high-strength steels during punching. Such studies would contribute to the development of more precise and adaptive punching processes, tailored to the specific requirements of advanced manufacturing applications.

In summary, this study demonstrates that punch shaft geometry, punching velocity, and punch-die clearance play pivotal roles in determining the shear zone height and, consequently, the quality of punched components. The adoption of a pointed punch design, combined with optimized process parameters, offers a robust solution for achieving high-quality punched edges with minimal defects. The findings not only advance the theoretical understanding of shear zone formation but also provide practical guidance for industrial applications, ensuring that punching processes can be optimized for precision, efficiency, and reliability.

## 4. Conclusions

This study contributes to the existing body of research on cold forming by focusing specifically on the evolution of the shear zone height during the punching of S500 MC high-strength steel sheet metal. While previous studies have explored various aspects of punching, this work provides a detailed analysis of how punching speed and punch-die clearance influence shear zone characteristics, thereby affecting the quality of the punched hole.

The key findings of this research are as follows:

The height of the shear zone is directly correlated with the quality of the punched hole. A well-defined shear zone ensures smoother edges, reduced burr formation, and improved dimensional accuracy, all of which are critical for high-precision applications.

The shape of the punching tool, punching speed, and punch-die clearance significantly impact the shear zone height and, consequently, the finished product quality. For a pointed punch shape, lower punching speeds are preferred, as they promote a more controlled deformation process. Conversely, for a simple shear punch design, higher speeds result in a more pronounced shear zone height in the slug.

This study identified that a pointed punch geometry enhances the stability of the shear zone height (ranging between 0.45 mm and 0.7 mm), reducing sensitivity to process variations. For a punch-die clearance of 0.25 mm, distinct peaks in shear zone height were observed at punching speeds of 3 mm/s, 7 mm/s, and 9 mm/s, with the optimal combination being a 0.25 mm clearance and a 9 mm/s speed. This combination maximizes shear zone height, ensuring superior edge quality and minimal defects.

The findings offer practical guidance for industrial applications, enabling operators—regardless of experience—to assess and optimize punching parameters for improved product quality. This study underscores the importance of tailoring punching conditions to specific materials and applications, as generic parameters from literature may not yield optimal results.

The results align with previous studies [22,25,27], which emphasize the role of punch geometry, velocity, and clearance in shear zone formation. However, this work extends current knowledge by demonstrating that a pointed punch design, combined with optimized parameters, yields a more stable and predictable shear zone height, particularly beneficial in high-volume production environments.

While this study provides valuable insights, further research is needed to validate these findings under real-world industrial conditions and for different materials, such as aluminum alloys or advanced high-strength steels. Future work could explore advanced numerical simulations, material microstructure effects, and real-time optimization using machine learning and AI to refine punching processes further.

In summary, this research highlights the critical role of punch geometry, speed, and clearance in determining shear zone characteristics and punched component quality. The adoption of a pointed punch design, combined with optimized process parameters, offers a robust solution for achieving high-precision, defect-free punching. These findings provide actionable insights for manufacturers, supporting the ongoing advancement of modern punching technology.

## Figures and Tables

**Table 1:** Elasticity parameters and density of AISI D2 and S500MC steel [15].

Parameters	AISI D2	S500MC
E (GPa)	230	209
ν	0.28	0.28
Density (kg/m^3^)	7.9 × 10^3^	7.9 × 10^3^
A (N/mm^2^)	1490	510
B (N/mm^2^)	660	220
n	0.04	0.28
C	0.29	0.0019
m	0.38	1
$\bar{\dot{\varepsilon 0}}$	1	1
D_1_	0.69103	0.53467
D_2_	0	0
D_3_	0	0
D_4_	−0.03524	−0.01913
D_5_	0	0

**Table 2:** Punch Geometries, Shear zone height, Speeds and Clearances.

Punch Geometry	Shear Zone Height	Punch Speed (mm/s)	Punch-Die Clearance (mm)
Blank punch	≤0.7	7	0.25
	≤0.7	9	0.3
	≤0.7	10	0.35
Pointed punch shape	0.45 to 0.7	7	0.25
	0.45 to 0.7	9	0.3
	0.45 to 0.7	10	0.35
Simple-shear shaft punch	0.5	3	0.25
	0.7	7	0.3
	0.7	9	0.35

## Data Availability

The original contributions presented in this study are included in the article. Further inquiries can be directed at the corresponding author.
